# MicroRNA-146a Overexpression Impairs the Positive Selection during T Cell Development

**DOI:** 10.3389/fimmu.2017.02006

**Published:** 2018-01-23

**Authors:** Zinan Li, Siya Zhang, Ying Wan, Menghua Cai, Weiqing Wang, Yuli Zhu, Zhen Li, Yu Hu, Huaishan Wang, Hui Chen, Lianxian Cui, Xuan Zhang, Jianmin Zhang, Wei He

**Affiliations:** ^1^Department of Immunology, Research Center on Pediatric Development and Diseases, Institute of Basic Medical Sciences, Chinese Academy of Medical Sciences and School of Basic Medicine, Peking Union Medical College, State Key Laboratory of Medical Molecular Biology, Beijing, China; ^2^Biomedical Analysis Center, Third Military Medical University, Chongqing, China; ^3^Department of Otolaryngology, Peking Union Medical College Hospital, Chinese Academy of Medical Science and Peking Union College, Beijing, China; ^4^Department of Rheumatology and Clinical Immunology, Peking Union Medical College Hospital, Chinese Academy of Medical Sciences and Peking Union Medical College, Beijing, China

**Keywords:** microRNA-146a, T cell homeostasis, T cell development, positive selection, Gimap4

## Abstract

MicroRNAs play crucial roles in modulating immune system. miR-146a, a potent feedback suppressor of NF-κB signaling, was shown to limit the innate immune response and myelopoiesis in a knockout mouse model. Here, we observed high lymphopoiesis demonstrated as mild splenomegaly and severe lymphadenopathy in a miR-146a transgenic mouse model. Overexpression of miR-146a resulted in enhanced proliferation and reduced apoptosis of T cells. More activated CD4^+^ T cells or effector memory T cells were observed in transgenic mice even under physiological conditions. Importantly, as one of the key steps to generate central tolerance, the positive selection of thymocytes is impaired in transgenic mice, resulting in more CD4^+^CD8^+^ double-positive thymocytes but fewer CD4^+^CD8^−^ and CD4^−^CD8^+^ single-positive thymocytes. The maturation of selected CD4^−^CD8^+^ thymocytes was also impaired, leading to more severe loss of CD4^−^CD8^+^ than CD4^+^CD8^−^ thymocytes in thymus of transgenic mice. Gene expression profiling analysis identified nine positive selection-associated genes, which were downregulated in transgenic mice, including genes encoding major histocompatibility complex class I/II molecules, IL-7 receptor α chain, and Gimap4, whose downregulation may contribute to the impairment of positive selection. Gimap4 was verified as a novel target of miR-146a. These findings further extend our understanding of the function of miR-146a in T cell biology and identify a novel regulatory mechanism underlying the positive selection during T cell development.

## Introduction

The immune system is an elaborately regulated network comprising many biological structures and processes to protect us from pathogens and malignancy. MicroRNA (miRNA), an RNA-based form of posttranscriptional regulation, has profound effects on hematopoietic development (1), immune cell differentiation, and the immune response (2). A series of loss-of-function studies have demonstrated a crucial role of the miRNA network in T cell differentiation and function by knocking out the key biosynthetic enzymes Drosha (3), DGCR8 (4), and Dicer (5, 6) in T cells or in certain T cell subsets.

miR-146a, the expression of which is mainly restricted to immune cells in the physiological context (7), has been proved to serve as a feedback suppressor for both the innate and adaptive immune responses by targeting several important adaptors or transcription factors (TFs), including IRAK1, TRAF6 (7–10), and STAT1 (11, 12) in the NF-κB and JAK-STAT signaling pathways. Loss-of-function studies on miRNA-146a knockout (KO) mice revealed a protective nature of miR-146a in some inflammatory settings such as bacterial or viral infection. Some autoimmune disorders, e.g., rheumatoid arthritis (RA) (13, 14), systemic lupus erythematosus (SLE) (15), and inflammatory bowel disease (9, 16) were also linked to ectopic miR-146a expression. Although most of the investigators believe that miR-146a serves as an beneficial factor (7, 17), there are still a few reserved views about its pathological role in autoimmune diseases such as RA (18), in which abnormally high levels of miR-146a are often observed at peripheral blood or lesion sites. In addition, most of the miRNAs exert biological multifunction due to the “one-to-many” mode of action (19). It is reasonable to assume that miR-146a shows different characteristics in some specific inflammatory settings. Thus, a gain-of-function study appears imperative to achieve a full picture of miR-146a in regulating the immune response.

With regard to hematopoietic processes, miR-146a also plays an indispensable role. It has been solidly proven that miR-146a serves as a guardian of hematopoietic stem cells (HSCs) at the physiological level by inhibiting myeloid differentiation and proliferation (20). miR-146a knockout mice show severely reduced HSC numbers in bone marrow, myeloid hyperplasia in peripheral blood, and even tumors in spleen (7, 8). Compared to myelopoiesis, less is known about the regulatory role of miR-146a in lymphopoiesis. To address this question, Starczynowski et al. analyzed the lymphopoiesis at early stage in bone marrow and found that enforced miR-146a overexpression impaired lymphopoietic process of hematopoietic stem/progenitor cells and finally reduced CD4^+^ T cell counts in peripheral blood (21). In addition, Kirigin et al. found that miR-146a fluctuates in thymocytes at different developmental stages in thymus (22). These findings indicate that miR-146a may act as a modulator during T cell development. Therefore, the systemic phenotyping of miR-146a in a mouse model is essential to gain some insights.

Thymocytes go through a series of defined and coordinated developmental steps in thymus and eventually mature into functional T cells. Generally, the process can be divided into four stages based on the expression of the co-receptor molecules CD4 and CD8 on the surface of thymocytes: double-negative (DN, CD4^−^CD8^−^) cells, double-positive (DP, CD4^+^CD8^+^) cells, CD4 single-positive (SP, CD4^+^CD8^−^), or CD8 SP (CD4^−^CD8^+^) cells. In the process of development, several events such as TCR rearrangement, positive and negative selection, will ensure that the developing thymocytes generate vast polyclonal TCRs with appropriate affinity to foreign but not self-antigens and finally develop into functionally competent T cells. In which, positive (23, 24) and negative selections (25, 26) are crucial for generating central tolerance. Disruption of these key steps will lead to an escape of autoreactive T cells into periphery and finally cause autoimmune pathology.

Guo et al. generated a miR-146a systemically expressed transgenic (Tg) mice and observed an autoimmune lymphoproliferative syndrome (ALPS)-like phenotype on this model, including enlarged spleens and lymph nodes (LNs), inflammatory infiltration in the livers and lungs, increased levels of DN T cells in peripheral blood, and increased serum immunoglobulin G levels. Dampened Fas expression by miR-146a and homeostatic proliferation of germinal center (GC) B cells were thought to be the major etiological factors, which lead to spontaneous GC formation in lymphoid nodes on this model (27). In our study, we used the same model but focused on aberrant changes in T cells and tried to reveal its pathological role in autoimmune disease. We observed abnormal subsets composition, imbalanced homeostasis, and disturbed development of T cells in miR-146a Tg mice. Like B cells in this model, the apoptotic and/or proliferative activity of T cells was dampened by miR-146a. In addition, the positive selection of thymocytes and the further maturation of CD4 and CD8 SP (especially CD8 SP) population are impaired in miR-146a Tg mice. miR-146a regulates positive selection during T cell development by targeting several genes, including the genes encoding major histocompatibility complex (MHC) class I/II molecules, the IL-7 receptor α chain, and Gimap4.

## Materials and Methods

### Mice

miR-146a Tg mice were gifts from Dr. Ying Wan at Third Military Medical University. The generation of this mouse model was described in detail in Guo et al.’s previous report (27); Balb/c mice serving as wild-type (WT) control were purchased from the Laboratory Animal Center of the Military Medical Sciences Academy. The first filial generation of Tg or WT mice was paired randomly to breed the second generations. The coming generations were used in this research. All mice were housed and bred in the barrier facility at Peking Union Medical College. The animal experiments were approved by the Institutional Animal Care and Use Committees of Peking Union Medical College.

### Cell Isolation

Mice were sacrificed by cervical dislocation, and then brachial, axillary, and inguinal LNs together with the thymus and spleen were placed in precooled HBSS. Organs were washed twice to remove blood and connective tissue.

A single-cell suspension was obtained from the LNs, spleen, and thymus by squeezing tissues through 40-µm cell strainers with HBSS. Suspension was centrifuged to remove the supernatant before resuspending the cells in complete culture medium as described in the following experiments. For splenic cells, an additional RBC lysis step was performed. Splenic cell suspension was centrifuged to remove supernatant. The sediment was resuspended in 1 mL ACK lysis and kept in room temperature. To stop the lysis, 10 mL FBS-free HBSS was added and then removed by centrifugation.

For thymic stromal cells, the incised thymus was placed into digestion medium composed of 0.15% (w/v) collagenase D and 0.01% (w/v) DNase I in RPMI 1640 medium containing 5% fetal calf serum. The thymus was then digested at 37°C on shakers (100 r/min). Every 10–15 min, the macroscopic fragments were allowed to settle under gravity, while the cell-containing supernatant was removed and saved on ice, followed by the replenishment of digestion media and next round digestion. The procedure was repeated until no macroscopic thymic fragments remained. All supernatant fractions were combined and centrifuged at 200 × *g* for 5 min at 4°C to obtain a pellet, which contained both thymocytes and stromal cells.

### Flow Cytometry (FCM)

To avoid non-specific staining, Fc blocker (BD Pharmingen, USA) was applied before staining. Cells from LNs and spleens were incubated with antibodies against CD3e (145-2C11), CD19 (6D5), the TCR β chain (H131), TCR γδ (GL3), CD4 (RM4-4), and CD8a (53-6.7) (BD Pharmingen, USA). Thymocytes were incubated with antibodies against CD4 (RM4-4), CD8a (53-6.7), CD25 (PC61), CD44 (IM7), CD62L (MEL-17), and CD69 (H1.2F3) (BD Pharmingen, USA). Thymic stromal cells were incubated with antibodies against MHC class I (34-1-2S) and II (M5/114.15.2) and CD127 (A7R34) (Biolegend, USA). Intracellular Bcl-2 (BCL/10C4) staining of thymocytes was performed according to the manufacturer’s instructions provided with the kit (Biolegend, USA). FCM was performed on a Gallios (Beckman Coulter, USA) or Accuri C6 (BD, USA) flow cytometer.

### Proliferation Assay

The proliferation of T cells induced by immobilized anti-CD3/28 was analyzed using a CFSE dilution assay as described previously. Briefly, splenic cells were stained with CFSE (a final concentration of 10 µmol/L in a cell suspension of 1 × 10^6^ cells/mL, Life Technologies, USA) and then stimulated with plate-coated anti-CD3/28 Abs (1 µg/mL each) for 48 h. CFSE dilution resulting from proliferation was analyzed with FCM. Staining for the surface markers CD3e (145-2C11) and CD8a (53-6.7) was also performed before FCM to distinguish CD4 (CD3^+^CD8^−^) and CD8 (CD3^+^CD8^+^) subsets.

### Apoptosis Detection

Splenic cells were resuspended in RPMI 1640 without FBS to induce apoptosis. After harvested at 48 or 96 h, cells were stained with 7-AAD and Annexin V (Biolegend, USA) together with antibodies against CD4 and CD8 (BD Pharmingen, USA). The level of apoptosis in both the CD4 and CD8 subsets was determined using FCM.

### RNA Sequencing

Total RNA was isolated from the thymus of WT and Tg mice using Trizol (Invitrogen, USA) according to the manufacturer’s instructions. The integrity of each RNA sample was verified on an Agilent Bioanalyzer 2100 (Agilent Technologies, USA). After purification using Dynabeads Oligo (dT) (Life Technologies, USA), 100 ng mRNA per sample was processed using NEB Next Ultra RNA Library Prep Kit for Illumina (NEB, USA) according to the manufacturer’s recommendations. The libraries were sequenced with an Illumina HiSeq 2500 (Illumina, USA). Sequence data were extracted in the FastQ format and used for mapping. Reads that passed quality filtering were mapped against the *Mus musculus* genome using HotHap2, and the only uniquely mapped reads were used for counting. Then, the read counts were used to calculate fragment per kilobase of exon per million fragment values for each sample. The *q* value was used to control false discovery rates for multiple hypothesis testing. Genes with a fold change over 2 and *q* < 0.01 were considered differentially expressed. Gene ontology (GO) analysis was performed using GoPipe (28), and Genes and Genomes (KEGG) pathway analysis was performed as described in the previous report (28, 29). The data discussed in this publication have been deposited in NCBI’s Gene Expression Omnibus and are accessible through GEO Series accession number GSE99428.

### Western Blot

Total protein extracts were prepared from the thymuses of both WT and Tg mice (6 weeks old, *n* = 3) and then loaded into each lane of a 10% SDS-PAGE gel. After electrophoresis, total proteins were transferred onto a nitrocellulose membrane, and Gimap 4 was detected using an antibody against Gimap4 (Santa Cruz, CA, USA). Beta-actin was used for normalization.

### Luciferase Assay

The vector containing the full-length gimap4 3′UTR (pMIR-REPORT Luciferase-gimap4, 20 ng/well) together with a plasmid encoding miR-146a (pCDH-CMV-miR-146a-EF1-copGFP, 40 ng/well) were co-transfected into 293T cells (in 24-well plate) with Lipofectamine 2000 (Invitrogen, USA). A plasmid constitutively expressing Renilla luciferase was also transfected to normalize for transfection efficiency. Luciferase activity was detected using the Dual-Luciferase Reporter Assay Kit (Promega, USA) according to the manufacturer’s instructions.

## Results

### miR-146a Tg Mice Show Splenomegaly and Lymphadenopathy Which Mimics the Symptom of ALPS

**Figure 1:**
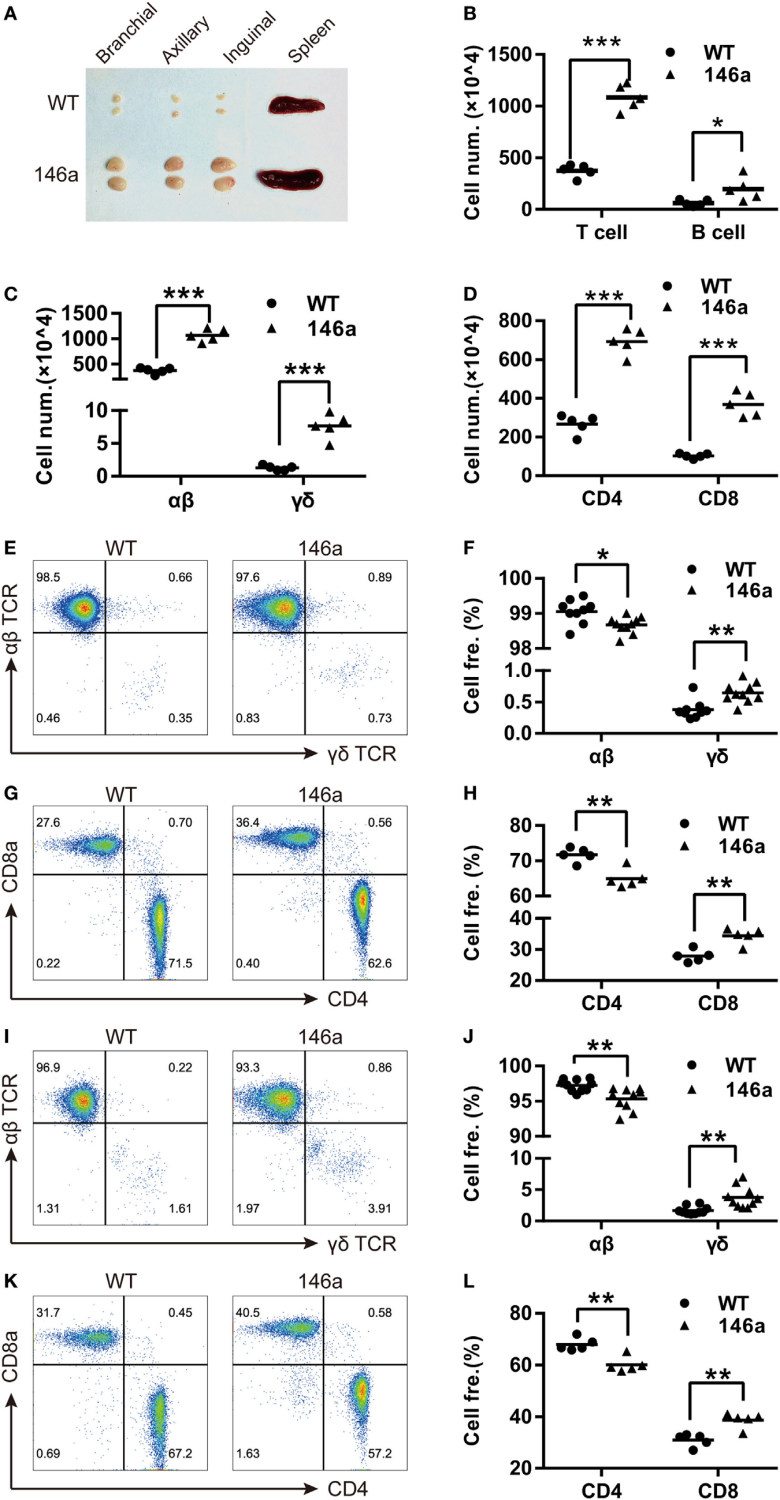
miR-146a transgenic (Tg) mice show splenomegaly and lymphadenopathy. **(A)** Bilateral inguinal, axillary, and brachial lymph nodes (LNs) and spleens were isolated from 7-week-old female miR-146a Tg (bottom) or wild-type (WT) (top) mice. **(B–D)** Flow cytometry (FCM) analysis of T cells and B cells **(B)**, αβ/γδ T cells **(C)**, and CD4^+^/8^+^ T cells **(D)** from the LNs of miR-146a Tg or gender- and age-matched WT mice. **(E–H)** FCM analysis of the proportions of αβ/γδ T cells **(E,F)** and CD4^+^/CD8^+^ T cells **(G,H)** in LNs from miR-146a Tg and WT mice. **(I–L)** FCM analysis of the proportions of αβ/γδ T cells **(I,J)** and CD4^+^/CD8^+^ T cells **(K,L)** in spleens from miR-146a Tg and WT mice. Each dot represents one mouse, and the values represent the mean ± SD. Statistical significance is indicated as follows: *P < 0.05, **P < 0.01, ***P < 0.001 (Holm–Sidak method).

A marked phenotype of miR-146a Tg mice is splenomegaly and lymphadenopathy (Figure 1A). Thus, we performed FCM to characterize the T and B cells in spleens and LNs from miR-146a Tg mice. The results showed that the absolute numbers of all lymphocyte subsets (B cells, T cells, and αβ/γδ or CD4^+^/CD8^+^ T cells) were significantly elevated by two- to five-fold in spleens or LNs from Tg mice relative to those from WT mice (Figures 1B–D). Compared to absolute count, the relative proportions of αβ T cells and CD4^+^ T cells were lower in Tg mice, and the relative proportions of γδ T cells and CD8^+^ T cells were significantly higher in both the LNs (Figures 1E–H) and spleens (Figures 1I–L) of Tg mice compared to WT mice. Taken together, these results indicate a directive role of miR-146a in T cell differentiation/polarization.

### miR-146a Overexpression Results in Elevated Frequency of T Cells with Activation or Memory Phenotype

**Figure 2:**
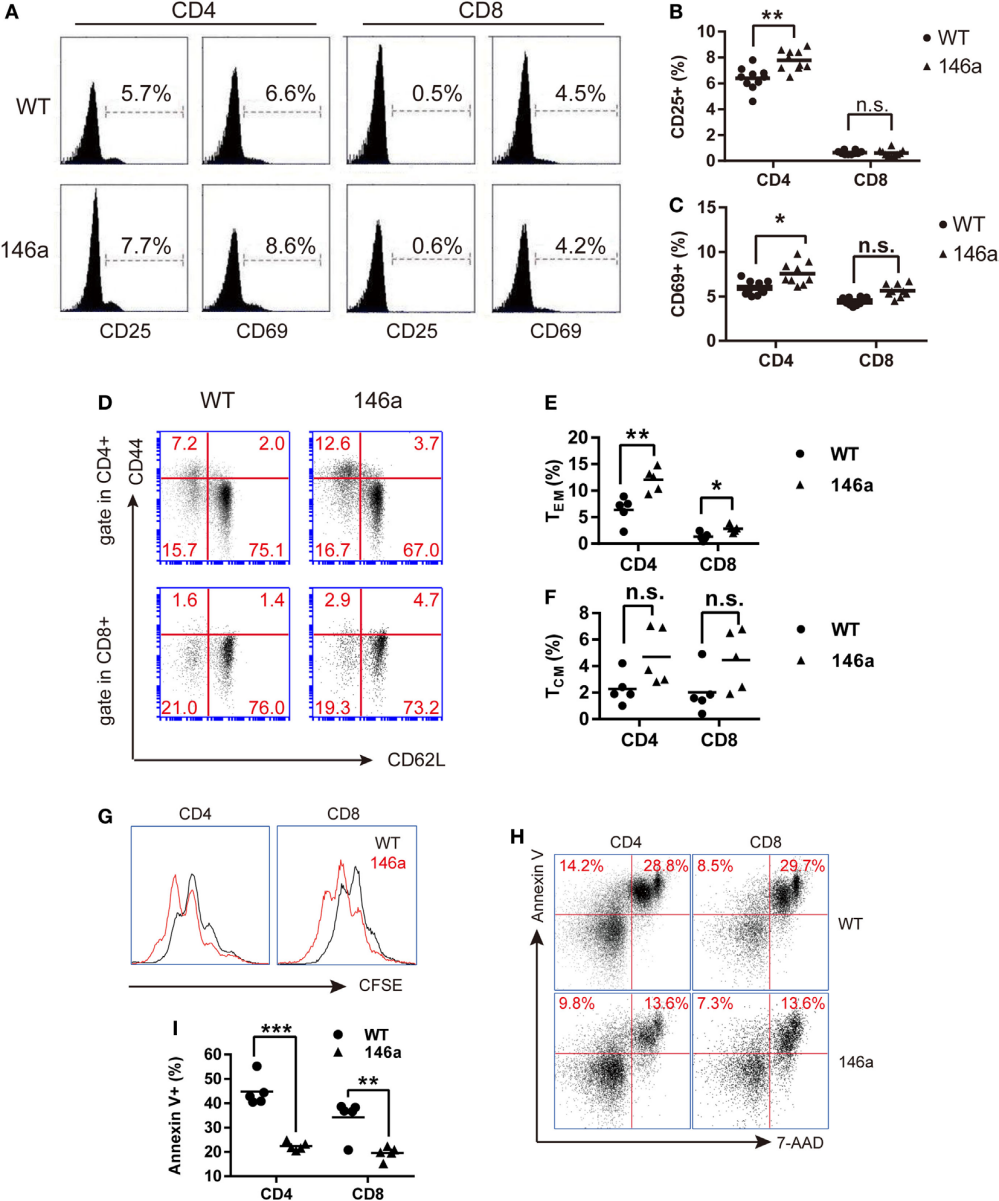
T cells from miR-146a transgenic (Tg) mice exhibit higher frequencies of activated or memory phenotype and exert higher proliferative but lower apoptotic activity. **(A–F)** Flow cytometry analysis of the proportions of CD4^+^ T cells or CD8^+^ T cells with an active CD25^+^/CD69^+^ phenotype **(A–C)** or with the memory phenotypes **(D–F)**, which is indicated as CD44^high^CD62L^−^ (T_EM_) or CD44^high^CD62L^+^ (T_CM_) in splenic cells from miR-146a Tg (*n* = 10) and gender- and age-matched wild-type (WT) (*n* = 10) mice. **(G–I)** Splenic T cells were isolated from miR-146a Tg and age-matched WT mice and then stimulated with immobilized anti-CD3/28 antibodies **(G)** or cultured in FBS-free RPMI 1640 medium **(H,I)**. The proliferation and apoptosis of both CD4^+^ T cells and CD8^+^ T cells were examined using CFSE dilution **(G)** and Annexin V/7-AAD staining **(H,I)**, respectively. Each dot in the plots represents one mouse, and the values represent the mean ± SD. Statistical significance is indicated as follows: *P < 0.05, **P < 0.01, and ***P < 0.001 (Holm–Sidak method).

In addition to the proportion changes in T cell subsets, we also examined the expression levels of the canonical T cell activation markers CD25, CD44, and CD69 and the negative marker CD62L on T cells using FCM. The result showed a slight increase in the CD25^+^ or CD69^+^ population of CD4^+^ T cells, but not CD8^+^ T cells, in Tg mice (Figures 2A–C), indicating that more CD4^+^ T cells are activated in miR-146a Tg mice, even under physiological conditions.

By using surface marker CD44 and CD62L, T cells could be divided into two specific memory T cell subsets: effector memory T cells (T_EM_) (CD44^high^CD62L^−^) and central memory T cells (T_CM_) (CD44^high^CD62L^+^) (30). We found that the proportion of T_CM_ and T_EM_ cells show uptrend in both the CD4^+^ and the CD8^+^ T cell populations from the LNs of miR-146a mice (Figures 2D–F), indicating that overexpression of miR-146a results in abnormal increases in the memory T cell population (especially T_EM_) *in vivo*.

### Overexpression of miR-146a Enhances Proliferation but Restrains Apoptosis in Peripheral T Cells

In addition to the cell number changes, we sought to determine whether the overexpression of miR-146a affects T cell function. To this end, we performed a CFSE dilution assay and Annexin V/PI staining to examine the proliferative and apoptotic activity of T cells *in vitro*. The results showed that peripheral CD4^+^ and CD8^+^ T cells from miR-146a Tg mice proliferated to a greater extent than CD4^+^ and CD8^+^ T cells from WT mice when stimulated with immobilized anti-CD3/28 antibodies (Figure 2G). In contrast, FBS starvation-induced apoptosis was significantly lower in CD4^+^ T cells and CD8^+^ T cells from miR-146a Tg mice than cells from WT mice (Figures 2H,I). Collectively, these results suggest that the homeostasis of T cells in Tg mice is disturbed by miR-146a overexpression.

### miR-146a Overexpression Impacts the Positive Selection As Well As the Further Maturation of CD4 and CD8 SP Thymocytes

**Figure 3:**
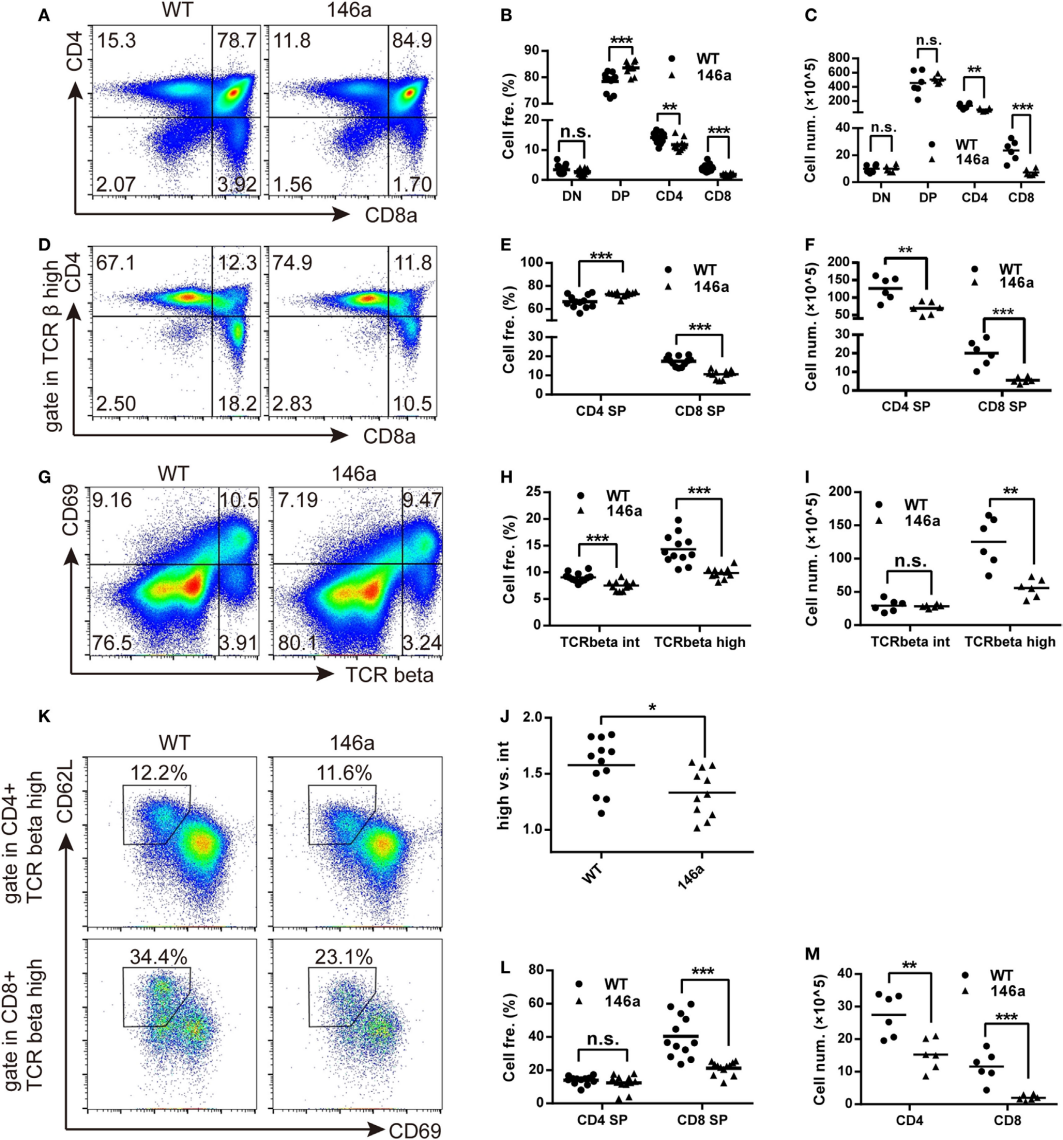
Positive selection and the further maturation of both CD4 and CD8 single-positive (SP) thymocytes are impaired in miR-146a transgenic mice. The frequency and absolute count of each thymocyte subsets were analyzed by flow cytometry (FCM). **(A–C)** Thymocytes were classified into three stages [double negative (DN), double positive (DP), or CD4/8 SP] based on the expression of the surface markers CD4 and CD8. Frequency **(B)** and absolute count **(C)** of thymocyte at each stage were analyzed by FCM. **(D–F)** Frequency **(E)** and absolute count **(F)** of positively selected (TCRβ^high^) CD4 and CD8 SP thymocytes were analyzed by FCM. **(G–J)** Frequency **(H)** and absolute count **(I)** of thymocytes undergoing positive selection, which is indicated as a transition from CD69^+^TCRβ^int^ to CD69^+^TCRβ^high^ were analyzed by FCM. The ratios of CD69^+^TCRβ^high^ (after selection) to CD69^+^TCRβ^int^ (before selection) were calculated **(J)**. **(K–M)** Maturation of selected CD4 and CD8 SP thymocytes were analyzed by FCM. Frequency **(L)** and absolute count **(M)** of thymocytes with a mature phenotype (CD69^−^CD62L^high^) were recorded. Each dot represents one mouse, and the values represent the mean ± SD. Statistical significance is indicated as follows: *P < 0.05, **P < 0.01, and ***P < 0.001 (Holm–Sidak method).

Flow cytometry analysis showed a mild increase in frequency of DP stage along with a decrease in the frequency of both CD4 and CD8 SP cells in miR-146a Tg mice compared to WT mice (Figures 3A,B). It indicates that the transition from DP stage to SP stage was impaired during T cell development in miR-146a Tg mice. A decrease of cell count of CD4 and CD8 SP (Figure 3C) further confirm our conclusion.

As we all know that the most important event in the DP stage of thymocytes is positive selection. CD4^+^CD8^+^T cell receptor^intermediate^ (TCR^int^) thymocytes interact with thymic stromal cells in a TCR-MHC class I/II-CD4/8-dependent manner and then transiently upregulate the expression of the surface marker CD69 and the TCR β chain (31, 32). The upregulation of the TCR β chain, as one of two core processes in positive selection, provides a strengthened TCR signal to selected thymocytes and rescue them from apoptosis (31). Therefore, we next analyzed the frequency and absolute count of CD69^+^TCRβ^int^ and CD69^+^TCRβ^high^ thymocytes. We found that both of the frequency and absolute count of CD69^+^TCRβ^high^ populations decreased in miR-146a Tg mice (Figures 3G–I). The ratio of CD69^+^TCRβ^high^ thymocytes to CD69^+^TCRβ^int^ thymocytes frequency was also reduced (Figure 3J). These results indicate that miR-146a overexpression inhibits the process of positive selection.

After a temporary stage identified as CD4^+^CD8^+^CD69^+^TCRβ^high^, thymocytes downregulate the CD4 or CD8 co-receptor, acquire a semimature phenotype of CD4^+^ (or CD8^+^) TCRβ^high^CD69^+^ heat-stable antigen (HSA)^high^Qa-2 (CD24)^low^CD62L^low^, and then further differentiate into a mature phenotype of CD4^+^ (or CD8^+^) TCRβ^high^CD69^−^HSA^low^Qa-2^high^CD62L^high^ (32). Thus, we examined the frequency and absolute count of thymocytes with mature phenotypes in the CD4 SP and CD8 SP populations. The results showed that the frequency of CD69^−^CD62L^+^ thymocytes was unchanged in the CD4 SP population but smaller in the CD8 SP population in miR-146a Tg mice (Figures 3K,L), while the absolute cell count decreased in both populations (Figure 3M). Compared with CD4 SP, the further maturation of CD8 SP population is more severely impaired, which is indicated by absolute count date (Figure 3F), and lead to a relatively higher frequency of CD4^+^ thymocytes (Figures 3D,E) in TCRβ^high^ population (dominant in selected thymocytes).

Taken together, these results suggest that miR-146a regulates positive selection during T cell development and inhibits the further differentiation of selected SP (especially CD8 SP) thymocytes from the semi-mature to mature phenotype.

### Several Genes Closely Related to Positive Selection Are Downregulated in the Thymus of miR-146a Tg Mice

To clarify the molecular mechanism through which miR-146a regulates T cell development, we performed next-generation high-throughput RNA sequencing to analyze the transcriptome profile in the thymus. One hundred and one differentially expressed genes were identified between WT and miR-146a Tg mice, 39 of which were classified into specific biological processes based on the annotations in the KEGG pathway database. The most impacted pathways with statistical significance were “cell growth and death,” “immune diseases,” and “metabolism of other amino acids.”

**Figure 4:**
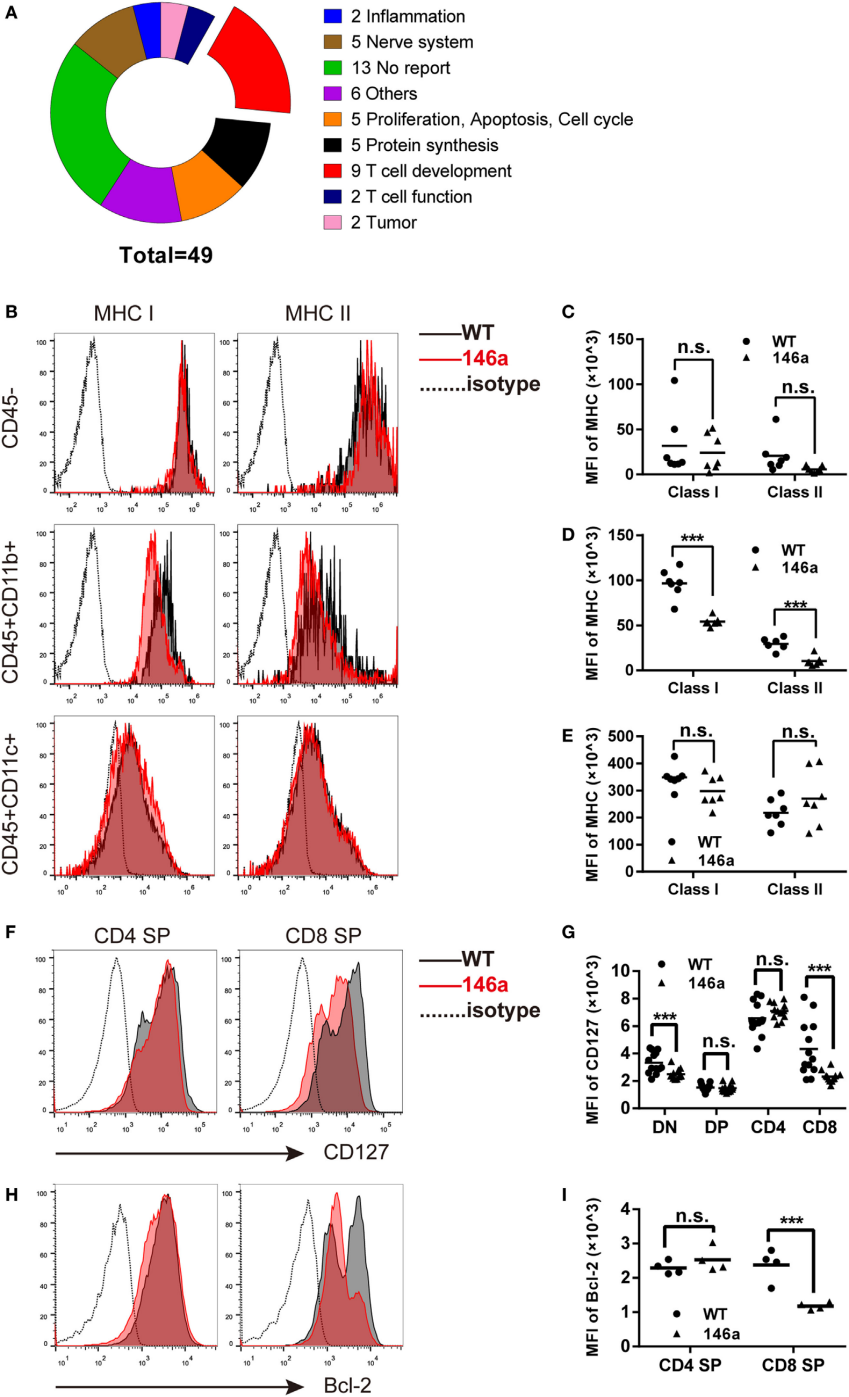
Global gene expression analysis of identified genes closely related to positive selection, in which major histocompatibility complex (MHC) molecules and CD127 are downregulated in thymic stromal cells and thymocytes, respectively. **(A)** Forty-nine downregulated genes were classified into nine groups based on their functions, in which nine (18.3%) genes are closely related to the positive selection process. **(B–E)** Thymic stromal cells were isolated from gender- and age-matched wild-type (WT) and transgenic (Tg) mice and then stained with anti-CD45, CD11b, CD11c, and MHC class I/II antibodies. The median florescence intensity (MFI) of MHC class I or II was determined in the CD45^−^, CD45^+^CD11b^+^, and CD45^+^CD11c^+^ population using flow cytometry (FCM). **(F–I)** Thymocytes were isolated from gender- and age-matched WT and Tg mice, and the expression (indicated by MFI) of CD127 **(F,G)** and Bcl-2 **(H,I)** was analyzed using FCM. Each dot represents one mouse, and the values represent the mean ± SD. Statistical significance is indicated as follows: *P < 0.05, **P < 0.01, *** and *P* < 0.001 (Holm–Sidak method).

Due to the negative regulatory effect of miRNAs on their targets, we further manually annotated a total of 56 downregulated transcripts covering 49 genes that could be the candidate targets of miR-146a by document retrieval and then classified them into nine groups based on their functions. The pie chart in Figure 4A shows that 9 of 49 (18%) genes were closely related to T cell development (Figure 4A). Importantly, all nine genes (*B2m, L1cam, gimap3, gimap4, H2-Ea-ps, Il7r, cd53, H2-K1*, and *Itm2a*) have been reported to participate in positive selection (Table 1). In addition, 10 genes (approximately 20%) are involved in proliferation/apoptosis or protein synthesis (Table S1 in Supplementary Material), which are also key cellular and molecular events during T cell development. The changes in transcription profile induced by miR-146a overexpression provide strong evidence supporting the observed effects on the process of T cell development and suggest several principal molecules as the candidate targets of miR-146a.

**Table 1:** Downregulated genes associated with T cell development.

Gene name	Fold change	Functional description	Reference
*B2m*	0.383	Component of major histocompatibility complex (MHC) class I molecules.	–
*L1cam*	0.155	Plays an important role in MHC class II-mediated peptide presentation in thymic epithelial cells, acting both in invariant chain degradation and in the generation of MHC class II-bound peptide ligands, which are presented by cortical thymic epithelial cells.	(33)
*gimap3*	0.479	Interacts with a group of Bcl-2 family members to regulate thymocyte survival and apoptosis.	(34)
*gimap4*	0.356	Closely related to IL-7R signaling. Interacts with Bax to accelerate the apoptosis of immature double-positive or other thymocytes.	(34)
*H2-Ea-ps*	0.448	Component of MHC class II molecules.	–
*Il7r*	0.418	Provides a survival signal to the selected thymocytes in both the early and late stages of T cell development.	(32, 35, 36)
*cd53*	0.322	Expressed on thymocytes depending on the signal provided by MHC molecules.	(37)
*H2-K1*	0.435	Component of MHC class I molecules.	–
*tm2a*	0.388	Expressed preferentially in the T lineage among hematopoietic cells and induced by MHC-mediated positive selection.	(38)

### miR-146a Tg Mice Show Reduced MHC Class I/II Molecules and the IL-7 Receptor α Chain on Thymic Stromal Cells or Thymocytes

As one of the most well-characterized events in T cell development, positive selection is generally believed to be driven by the interaction between MHC class I/II molecules on thymic stromal cells and TCRs together with CD4/8 molecules on thymocytes (39). At the same time, the signals provided by cytokine microenvironment are also crucial for the survival and further maturation of thymocytes (32, 35). Following the evidence generated by RNA-sequencing data, we first analyzed the expression of MHC class I/II molecules on CD45^−^ (enriched in epithelial and endothelial cells as well as fibroblasts) [Figure 4B (top) and Figure 4C], CD45^+^CD11b^+^ (enriched in macrophages) [Figure 4B (middle) and Figure 4D], and CD45^+^CD11c^+^ (enriched in dendritic cells) [Figure 4B (bottom) and Figure 4E] cells using FCM. The results showed a distinct decrease in both MHC class I and II molecules on CD45^+^CD11b^+^ cells and a downtrend in CD45^+^CD11c^+^ or CD45^−^ cells (except MHC class II in CD45^+^CD11c^+^).

Next, we analyzed the expression of CD127, the α chain of the IL-7 receptor, on thymocytes by FCM. The result showed that CD127 was downregulated in CD8 SP cells but not in the CD4 SP population (Figures 4F,G). In accordance with the change in CD127 expression, the level of the survival-promoting transcriptional factor Bcl-2 was also decreased in the CD8 SP population, while no significant change was observed in the CD4 SP proportion (Figures 4H,I).

Collectively, these results further identify the intrinsic and extrinsic molecules that drive the process of positive selection of thymocytes and might be direct or indirect targets of miR-146a.

### miR-146a Directly Targets *gimap4* mRNA

**Figure 5:**
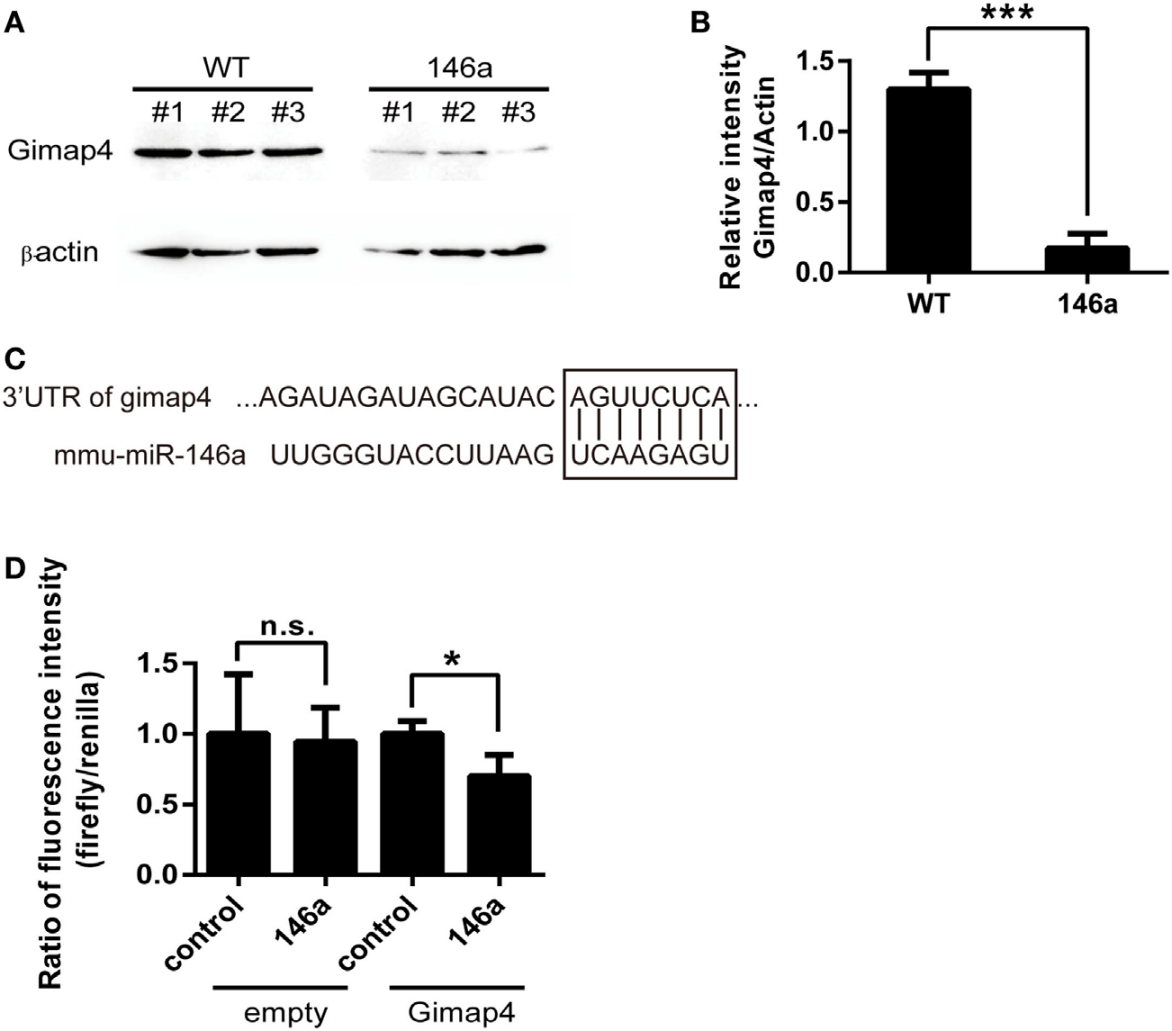
*Gimap 4* is a novel target of miR-146a **(A,B)**. Thymocyte proteins were extracted from gender- and age-matched wild-type (WT) and transgenic (Tg) mice and detected by Western blot with anti-Gimap4 antibodies **(A)**. The significant downregulation of gimap4 in Tg mice can be observed as an obvious decrease in the normalized intensity of bands **(B)**. **(C)** The predicted interaction between the 3′UTR of gimap4 mRNA and miR-146a. The seed region and the binding site are shown in the box. **(D)** A dual-luciferase assay confirmed that miR-146a downregulated the expression of gimap4 by targeting the 3′UTR of its mRNA.

Finally, by comparing the list of downregulated genes from the results of RNA sequencing and the list of miR-146a targets predicted by miRanda, miRWalk, or TargetScan, we identified one overlapping gene, *gimap4*. Western blot analysis confirmed that the expression of gimap4 was downregulated in the thymus of miR-146a Tg mice (Figures 5A,B). A dual luciferase reporter assay also indicated that miR-146a could directly inhibit the expression of gimap4 by targeting its 3′UTR (Figures 5C,D).

## Disscussion

On the basis of the Guo et al.’s study, we further investigated the physiological and pathological role of miR-146a in aspect of T cell. We performed systemic phenotyping on the miR-146a Tg mouse model and focused on the changes in T cells. Lymphadenopathy and splenomegaly, which are one of the clinical manifestations of ALPS patients, were also observed by us in this model. In secondary lymphoid organs, the changes on the proportion of CD4/CD8, αβ/γδ T cell-subsets indicate the aberrant immunity status. CD4^+^ and CD8^+^ T cells from Tg mice exhibited a higher frequency of activated phenotype, enhanced proliferative activity, and restrained apoptosis *in vitro*. Most of these characteristics in T cells are shared by many autoimmune diseases, e.g., SLE or RA, and indicate a pathological role of miR-146a in wide range of autoimmune diseases.

T cell development in thymus is essential in preventing autoimmunity by generating T cells carrying TCRs with appropriate affinity to foreign but not self-antigens. During this process, immature T cells must undergo positive and negative selection to acquire central tolerance. If the selection is disrupted, autoreactive T cell wills escape from thymus and contributes to break B cell tolerance in periphery (23). Thus, we next examined the T cell development in miR-146a Tg mice.

Defects in thymic T cell development were also observed in miR-146a Tg mice. Positive selection at the DP stage and the further maturation of CD4 and CD8 SP cells were impaired by the overexpression of miR-146a, which ultimately caused reductions in both CD4 and CD8 SP cells (especially the CD8 SP population) in the thymus. Transcriptome analysis provided us with more evidence to understand the regulatory mechanism of miR-146a on T cell development. By using high-throughput RNA-sequencing analysis, we identified nine T cell development-related genes that were downregulated by miR-146a in Tg mice. All of these genes are closely related to the positive selection of DP thymocytes. In addition, these nine genes can be divided into two groups based on their modes of action, i.e., “intrinsic” and “extrinsic.” The intrinsic genes, such as *il7*α and *gimap3/4*, are supposed to be expressed by thymocytes and act as receptors or adaptors in specific signaling pathways controlling cell fate such as apoptosis and survival, whereas the extrinsic genes such as major histocompatibility antigen (MHC) class I and II molecules are mainly expressed by thymic stromal cells, including thymic epithelial/endothelial cells, fibroblasts, thymic macrophages, and dendritic cells, and provide survival signals to thymocytes by interacting with pre-TCRs.

During positive selection, MHC molecules provide the signals that determine the fate of DP thymocytes as CD4 or CD8 SP cells (40). In addition, environmental cytokine signals such as IL-7 also provide survival signals or promote further differentiation to the selected thymocytes (32, 35). We confirmed the downtrend of both of MHC-I and MHC-II molecules in the CD45^−^ (enriched in epithelial and endothelial cells as well as fibroblasts), CD45^+^CD11b^+^ (enriched in thymic macrophages), and CD45^+^CD11c^+^ (enriched in dendritic cells) population (except MHC class II molecule in CD45^+^CD11c^+^ population). IL-7 provides a survival signal to thymocytes, especially CD4 and CD8 SP thymocytes and, to a greater extent, cells at the DN stage due to the relatively higher level of expression on these cells, throughout the entire process (41). In this study, we found that the α chain of the IL-7 receptor was downregulated on CD8 SP thymocytes, and, as a result, the level of the survival-promoting TF Bcl-2 was decreased, which subsequently impaired the maturation of CD8 SP thymocytes in miR-146a Tg mice.

*Gimap3* and *gimap4* belong to the *Gimap* (GTPase of the immunity-associated protein) family, which is also called the *IAN* (immune-associated nucleotide-binding protein) family and encodes functionally unknown GTP-binding proteins expressed in immune tissues (34, 42). Gimap3/4 levels increase in accordance with the positive selection of immature DP thymocytes to mature SP thymocytes, including during the early events in the transition from CD69^low^ DP cells to CD69^high^ DP cells and the late event in transition from CD69^high^ CD4 SP cells to CD69^low^ CD4 SP cells (34). In addition, *gimap3* and *gimap4* act antagonistically during the apoptosis of thymocytes. Overexpression of *gimap4* causes the apoptosis of DP thymocytes and then impairs subsequent T cell development, while *gimap3* provides a survival signal to selected CD4^+^ and CD8 SP cells (34). The regulation of *Gimap3* and *Gimap4* expression by miR-146a may control the balance between the apoptosis and survival of thymocytes at the DP stage and subsequently regulate positive selection.

Interestingly, *gimap4* was predicted to be the target of miR-146a by several software packages, including miRanda, miRWalk, and TargetScan. Western blot analysis confirmed that *gimap4* was downregulated in the thymocytes of miR-146a Tg mice. We also proved that miR-146a directly interacts with the 3′UTR of *gimap4* mRNA using a luciferase reporter assay. Nevertheless, it seems that the downregulation of gimap4 cannot explain the effect of miR-146a on T cell development, since *gimap4* KO mice show no obvious defect in T cell development (43).

It is noteworthy that the apoptosis of T cells in peripheral and thymocytes in central lymphoid organs are affected by miR-146a, but the consequence goes opposite. In thymus, miR-146a promotes apoptosis of CD8 SP, probably, by downregulating IL-7R α chain, whereas in peripheral lymphoid organs, miR-146a inhibits apoptosis of both CD4 and CD8 T cells by unknown mechanism. A previous study by others reported that miR-146 inhibited the apoptosis of Jurkat cells, a human T cell leukemia cell line, by targeting gene encoding pro-apoptotic protein FADD (44). We suppose that miR-146a regulates the apoptotic activity of peripheral T cell in the same way. To address this question, a systemic examination on pro- and anti-apoptotic effector molecules such as caspase 3 should be performed.

In summary, our findings strongly suggest that miR-146a participates in regulating the positive selection during T cell development in thymus and the T cell homeostasis in periphery. miR-146a overexpression results in defective T cell development of both CD4^+^ and CD8^+^ T cells and an autoimmune-like T cell disorder in periphery. Our findings further extend our understanding of the biological function of miR-146a and identify a novel mechanism underlying positive selection during T cell development.

## Author Contributions

ZL designed and performed experiments, analyzed data, and wrote the manuscript. SZ set up the breeding of animals and performed flow cytometry and cell culture. MC performed cell counting experiment. YZ performed the real-time PCR. YW assisted with setting up the breeding of animals. WW and ZL assisted with the analysis of the RNA-sequencing data. YH, HW, and HC assisted with flow cytometry. XZ and LC assisted with designing the scheme of this study. JZ and WH designed the scheme of this study and revised the manuscript.

## Conflict of Interest Statement

The authors declare that the research was conducted in the absence of any commercial or financial relationships that could be construed as a potential conflict of interest.
