# Supplementary material for: MicroRNA-146a Overexpression Impairs the Positive Selection during T Cell Development
Source: Front Immunol. 2018 Jan 23;8:2006. doi: 10.3389/fimmu.2017.02006 (PMC5787067; doi:10.3389/fimmu.2017.02006)
Supplement: Supplementary file 1 [file Image_1.PDF]

Supplementary Material

MicroRNA-146a Regulates Positive Selection During T Cell Development in mice

Zinan Li, Siya Zhang, Ying Wan, Menghua Cai, Weiqing Wang, Yuli Zhu, Zhen Li, Yu Hu, Huaishan Wang, Hui Chen, Lianxian Cui, Xuan Zhang, Jianmin Zhang*, Wei He*

*** Correspondence:**
Jianmin Zhang or Wei He
jzhang42@163.com or heweingd@126.com

A B

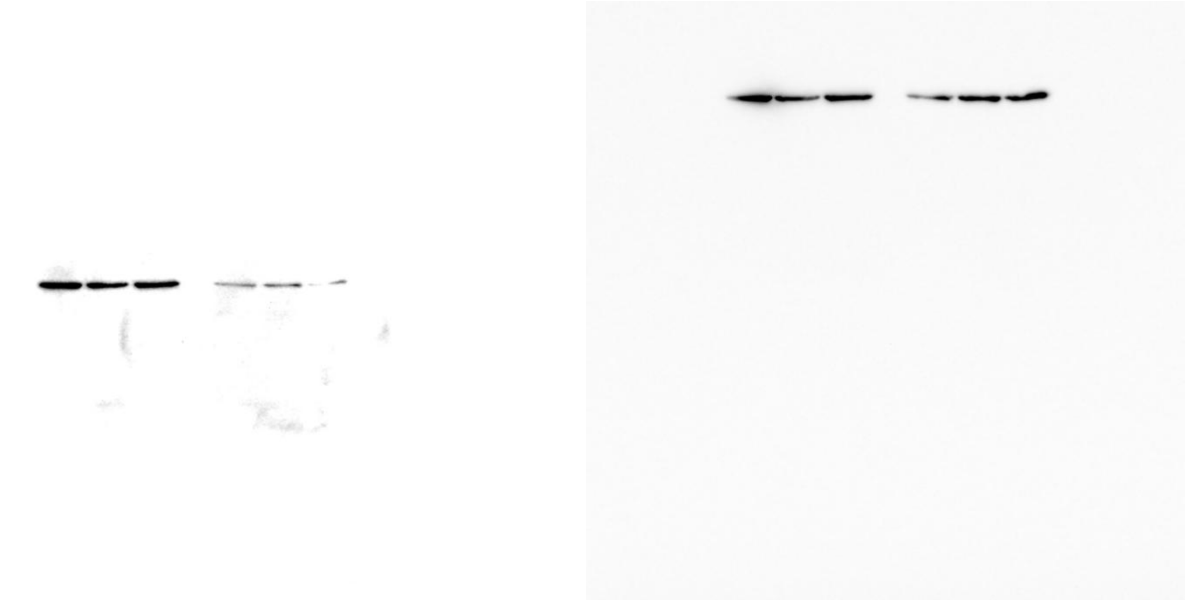

Supplementary Figure 1. Original graph of Western blot in Figure 5. (A) Gimap 4. (B) beta-actin
